# Artemether attenuates LPS-induced inflammatory bone loss by inhibiting osteoclastogenesis and bone resorption via suppression of MAPK signaling pathway

**DOI:** 10.1038/s41419-018-0540-y

**Published:** 2018-04-27

**Authors:** Haobo Wu, Bin Hu, Xiaopeng Zhou, Chenhe Zhou, Jiahong Meng, Yute Yang, Xiang Zhao, Zhongli Shi, Shigui Yan

**Affiliations:** 1 Department of Orthopedic Surgery, Second Affiliated Hospital, School of Medicine, Zhejiang University, Hangzhou, 310009 China; 2 Orthopedic Research Institute of Zhejiang University, Hangzhou, 310009 China

## Abstract

Osteolysis is an osteolytic lesion featured by enhanced osteoclast formation and potent bone erosion. Lacking of effective regimen for treatment of the pathological process highlights the importance of identifying agents that can suppress the differentiation and function of osteoclast. Artemether is a natural compound derived from Artemisia annua L. and it is popularized for the treatment of malaria. In present study, we demonstrated that artemether could suppress RANKL-induced osteoclastogenesis and expression of osteoclast marker genes such as tartrate-resistant acid phosphatase, cathepsin K, matrix metalloproteinase 9, nuclear factor of activated T-cell cytoplasmic 1, and dendritic cell-specific transmembrane protein. It inhibited the osteoclastic bone resorption in a dose-dependent manner in vitro. Furthermore, artemether attenuated RANKL-induced MAPKs (ERK, JNK, p-38) activity. In addition, we have showed that artemether was able to mitigate bone erosion in a murine model of LPS-induced inflammatory bone loss. Taken together, these findings suggest that artemether reduces inflammatory bone loss via inhibition of MAPKs activation during osteoclast differentiation, and it might be a potential candidate for the treatment of osteoclast-related disorders.

## Introduction

Total joint arthroplasty is now a routine orthopedic procedure for pain relief and mobility restoration to patients with end-stage joint diseases. However, inflammatory bone loss as a result of aseptic loosening and/or periprosthetic infection still remains tricky to deal with as a complication of joint arthroplasty^1^. Inflammatory reaction associated with prosthetic wear particles and/or bacterial products could activate immune system and procedure cytokines to enhance osteoclast recruitment and activity, and finally promote the dominance of bone resorption over bone formation, leading to bone destruction in the periprosthetic region^2^. The disrupted balance also contributes to other skeletal disorders such as rheumatoid arthritis and osteoporosis, which are characterized by an increase in the number and activity of osteoclasts and excessive bone loss^3^. Study has proven that adherent endotoxin contributes to wear particle-induced osteolysis by stimulating cytokine production and osteoclast differentiation^4^. Unfortunately, treatment strategy for inflammatory bone loss is still limited.

Osteoclasts are multinucleated giant cells belonging to the monocyte-macrophage lineage, which play a critical role in the physiological bone remodeling and pathological bone loss^5–7^. Macrophage-colony-stimulating factor (M-CSF) and receptor activator of nuclear factor-κ B ligand (RANKL) are key factors that involved in the survival and differentiation of osteoclasts. M-CSF binds to the colony stimulating factor 1 receptor on osteoclast precursors and keeps their survival and proliferation by activating ERK and PI3K/Akt^8^. Differentiation of osteoclast consists of several stages such as formation of tartrate-resistant acid phosphatase (TRAP) positive cells, fusion into multinucleated cells, activation to bone resorption, and spontaneous apoptosis^9,10^. RANKL is an essential cytokine during formation and activation of osteoclasts^11^. The interaction between RANKL and its receptor RANK recruits adaptor molecules TNF receptor-associated factors (TRAFs) and then activates several downstream signal pathways, including MAPKs, NF-κB, PI3K/Akt, followed by the activation of transcription factors like activator protein 1 (AP-1) and nuclear factor of activated T-cell cytoplasmic 1 (NFATc1)^12,13^. Activation of these downstream factors triggers osteoclast differentiation and function by inducing specific genes, including TRAP, cathepsin K (CTSK), matrix metalloproteinase 9 (MMP-9), and ultimately resulting in the formation of mature osteoclasts^12,13^.

Evidence has shown that osteoclastogenesis could be promoted by proinflammatory cytokines including TNF-α and IL-1^14^, and osteoclast overactivation plays a critical role in the imbalance between osteogenesis and osteoclastogenesis^15^. Thus, the suppression of osteoclastogenesis through inhibition of related inflammation could be a reasonable strategy for treatment of inflammatory bone loss.

In recent years, natural compounds have been an area of interest for seeking potential candidates of inhibiting osteoclastogenesis and bone resorption^15,16^. Here we identify artemether could be a potential candidate for treatment of LPS-induced inflammatory bone loss. Artemether is an antimalarial drug derived from artemisinin, which was originally extracted from Artemisia annua L^17^. Its commercial combination with lumefantrine has been proven to be an effective pharmacological regimen for treatment of malaria^18^. It has recently been found to have inhibitory effects on neuroinflammation of BV2 microglia, through modulating NF-κB and p38 MAPK pathway^19^. Given the importance of the two pathways in osteoclastogenesis and the anti-inflammatory effects of artemether^20^, we hypothesized that artemether might be a novel candidate for treatment of inflammatory bone loss through inhibition of osteoclast formation. Lipopolysaccharide (LPS) is a potent endotoxin that has been identified as an inducer of the immune system and a critical factor involved in the development of osteolytic bone loss^21^. Therefore, the purpose of this study was to investigate the effect of artemether in a mouse model of LPS-induced inflammatory bone loss and its effect as well as underlying mechanism during osteoclastogenesis.

## Results

### Artemether inhibited RANKL-induced osteoclast formation in vitro without inducing apoptosis

**Fig. 1:**
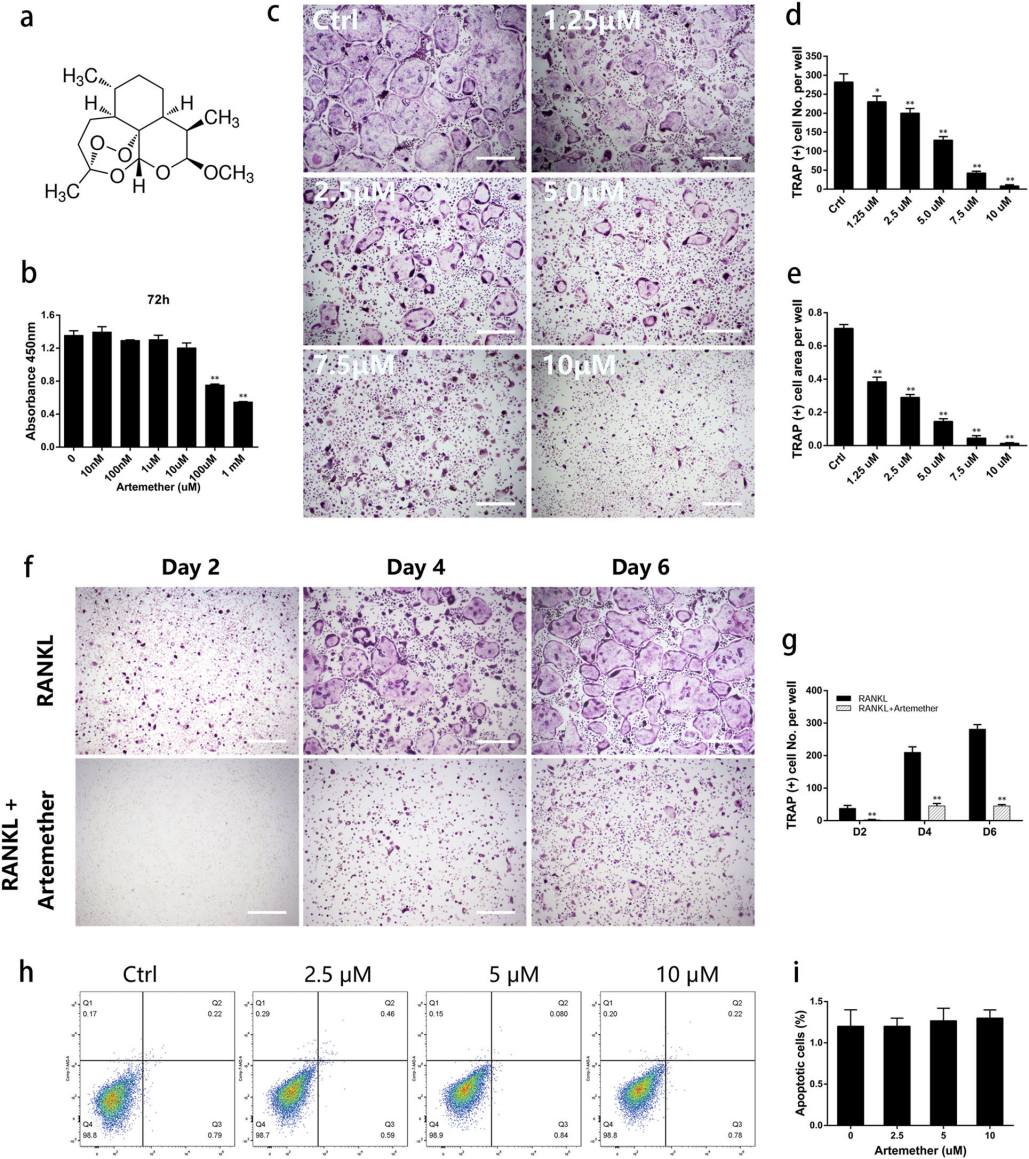
Artemether inhibited RANKL-induced osteoclastogenesis in a dose- and time-dependent manner in vitro without cytotoxicity. **a** The structure of artemether. **b** Cell counting kit-8 assay were performed to determine cell viability after treated by a series concentration of artemether for 72 h. **c**–**e** BMMs were cultured in induction medium with various concentration of artemether (0, 2.5, 5, 10 μM) for 5–6 days and TRAP staining was performed to visualize osteoclast formation. **f**, **g** BMMs were culture in induction medium with or without 10 μM artemether and stained with TRAP on day 2, 4, and 6, respectively. **h**, **i** Apoptosis of BMMs was determined by flow cytometry after treated with artemether for 72 h. All the data were confirmed by three repeated tests. The data were expressed as mean ± SD. *p < 0.05 and **p < 0.01 vs. the control group. Scale bar = 200 μm

The cytotoxicity of artemether on osteoclast precursor cells (BMMs) was determined by CCK8 assays and the IC50 value was 44 μM at 72 h (Fig. 1a, b). This indicated that artemether may partially attenuate the proliferation of BMM cells at high concentrations.

To investigate the effect of artemether on osteoclastogenesis, BMMs were cultured in complete α-MEM with M-CSF (25 ng/ml), RANKL (100 ng/ml), and artemether (0, 2.5, 5, and 10 μM) during osteoclast differentiation. BMMs without exposure to artemether (0 μM) differentiated into characteristic TRAP-positive multinucleated cells. However, those exposed to artemether (2.5, 5, and 10 μM) exhibited a decreased number of TRAP-positive multinucleated cells in a dose-dependent manner (Fig. 1c). Quantification indicated that mature osteoclasts number decreased in the presence of artemether from 282 (0 μM) to 8 (10 μM) per well and the area of mature osteoclasts reduced from 71 (0 μM) to 1% (10 μM) per well (Fig. 1d, e). Inhibition of artemether on osteoclastogenesis was also observed in a time-dependent manner (Fig. 1f,g). The formation of osteoclast was almost completely suppressed at high concentration (10 μM) of artemether, whereas the survival of BMMs were not affected at same concentration (Fig. 1h, i), indicating that these concentrations of artemether could attenuate osteoclast formation without interfering cell viability.

### Artemether suppressed RANKL-induced osteoclast-specific gene expression

**Fig. 2:**
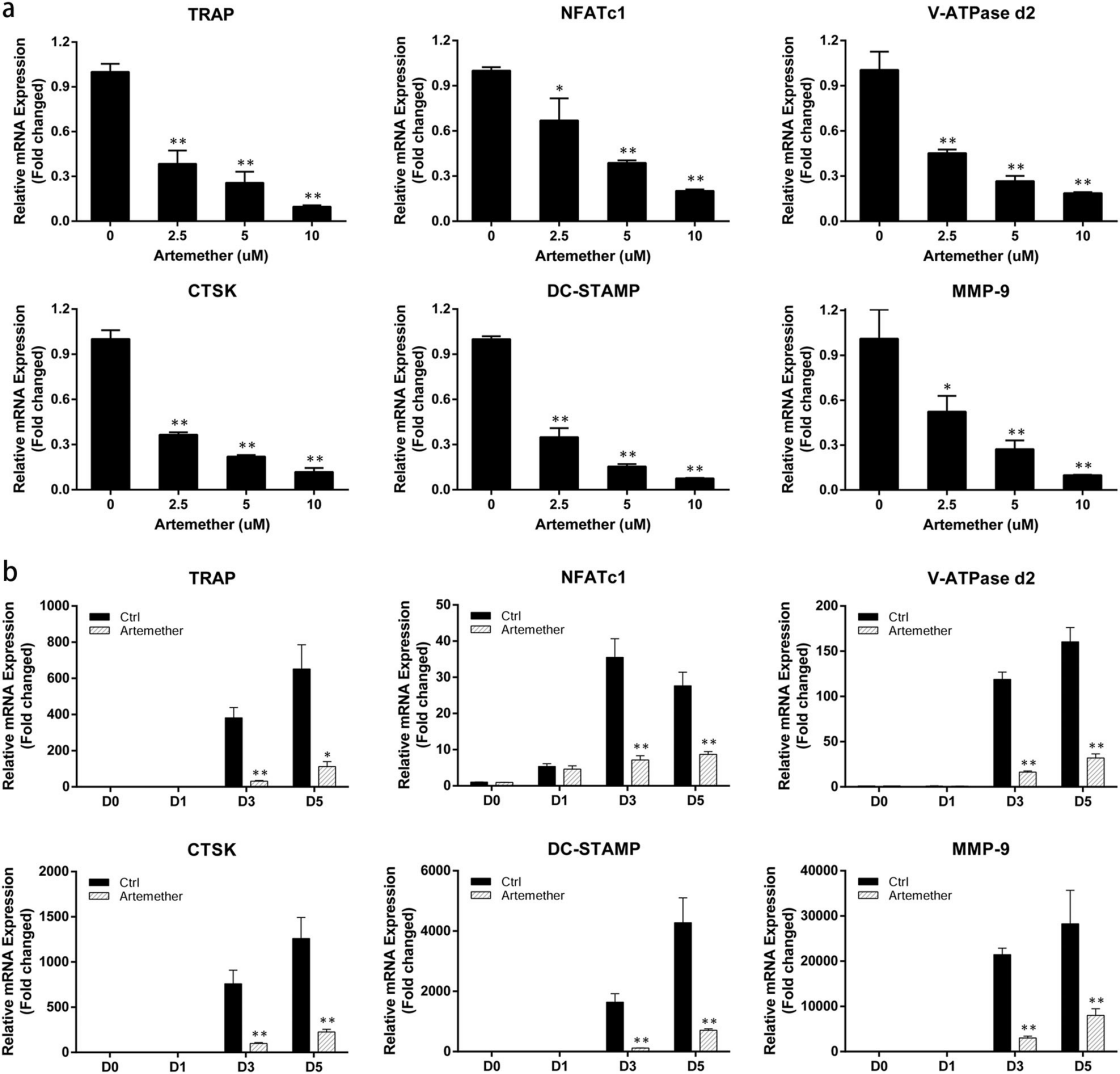
Artemether suppressed mRNA expression of RANKL-induced osteoclast marker genes including TRAP, NFATc1, V-ATPase d2, CTSK, DC-STAMP and MMP-9. **a** BMMs were cultured in induction medium for 5 days with various concentration of artemether, the mRNA expression of marker genes was determined qPCR at day 5. **b** BMMs were culture in induction medium with or without 10 μM artemether, and the mRNA expression of marker genes at day 0, 1, 3, and 5 was quantified by qPCR. All the data were confirmed by three repeated tests. The data were expressed as mean ± SD. *p < 0.05 and **p < 0.01 versus the control group

Expression of a specific set of genes was upregulated during differentiation of osteoclast^12^. The inhibition of artemether on RANKL-induced osteoclast-related mRNA expression (including TRAP, NFATc1, V-ATPase-d2, CTSK, dendritic cell-specific transmembrane protein/DC-STAMP, and matrix metalloprotein-9/MMP-9) was determined by quantitative PCR. Compared with control group, the RANKL-induced osteoclast-related gene expressions were strongly suppressed in the presence of artemether in a dose- and time-dependent manner (Fig. 2a, b). The relative gene expression decreased by 80% (NFATc1) to 92% (DC-STAMP) at high concentration (10 μM) of artemether. Collectively, the results further confirmed that artemether can attenuate osteoclast formation and its related gene expression during differentiation.

### Artemether impaired osteoclastic bone resorption

**Fig. 3:**
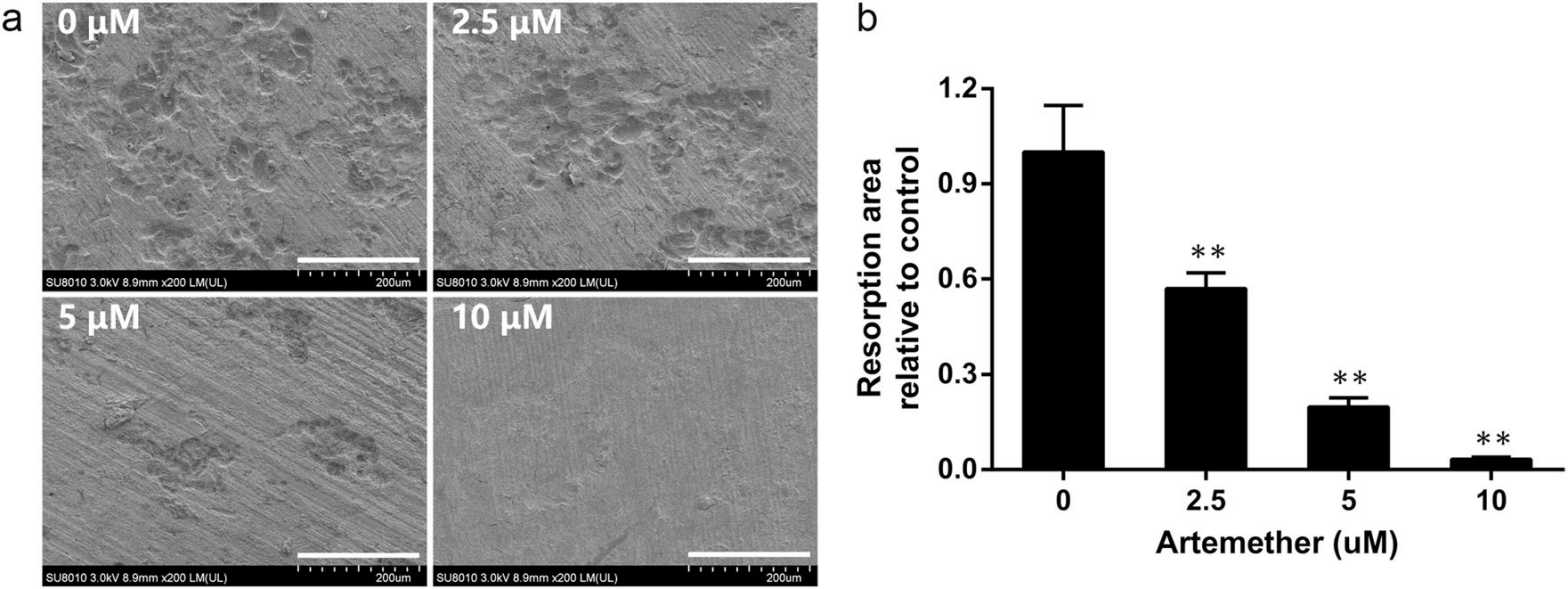
Artemether impaired osteoclastic bone resorption. **a** BMMs were seeded on bovine bone discs and cultured in induction medium with indicated concentration of artemether, and scanning electron microscope images of bone resorption pits were taken after mature osteoclast formed. **b** Quantification of bone resorption area in each group. All the data were confirmed by three repeated tests. The data were expressed as mean ± SD. **p < 0.01 vs. the control group. Scale bar = 200 μm

We next evaluated the efficiency of artemether on inhibition of osteoclastic bone resorption in vitro. Given that artemether could inhibit osteoclast formation, we speculated that osteoclastic bone resorption would also be suppressed. BMMs were seeded onto bone discs in induction medium with exposure to 0, 2.5, 5, and 10 μM of artemether. The results indicated that the bone resorption was active in vitro (0 μM). Percentage of resorption area decreased after exposure to 2.5 μM of artemether, and the resorption was almost completely inhibited at high concentration (Fig. 3a, b). Collectively, the result suggested that administration of artemether reduced the severity of bone resorption in vitro.

### Artemether attenuated RANKL-induced MAPK signaling pathways

**Fig. 4:**
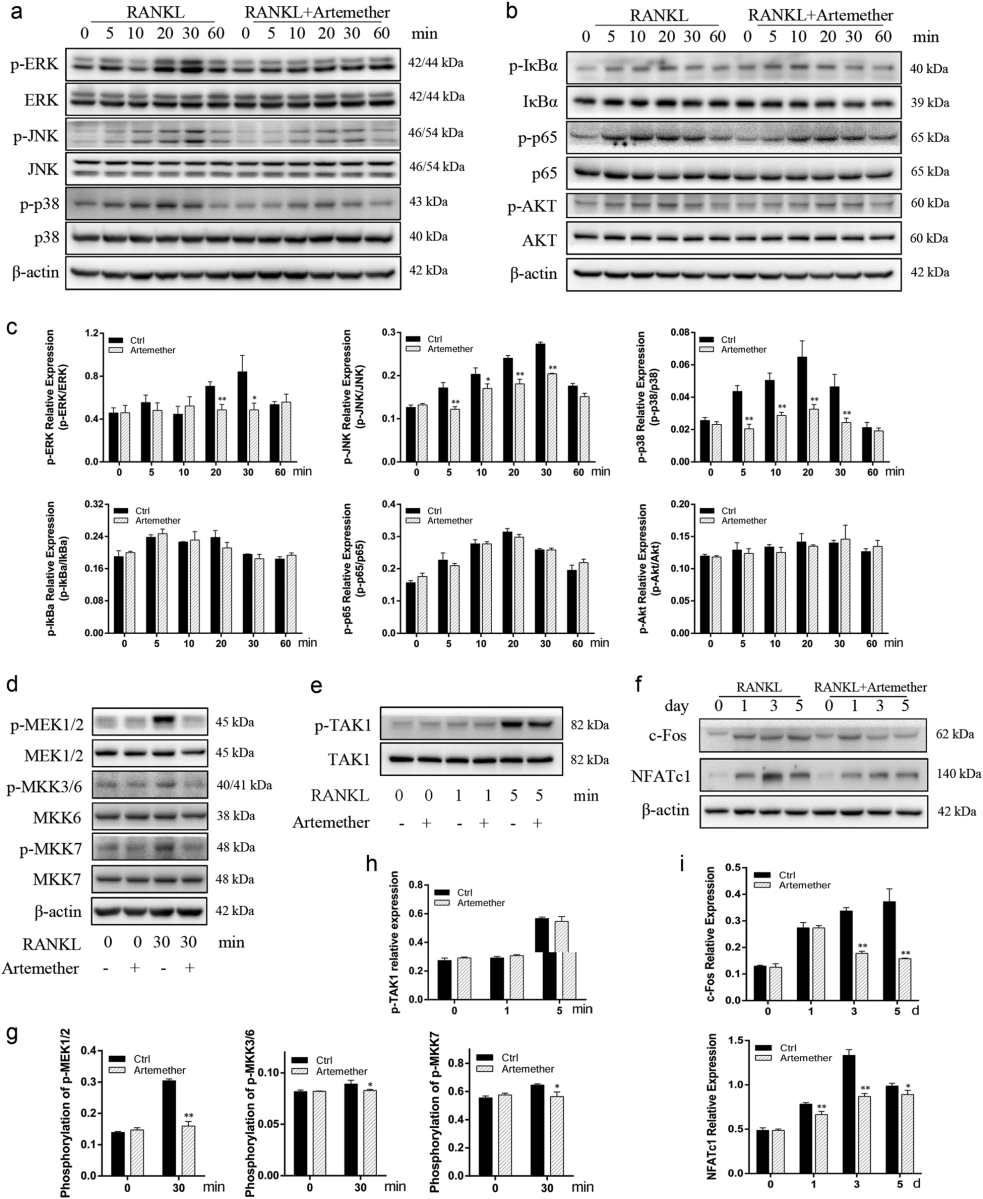
Artemether attenuated osteoclastogensis via inhibition of MAPK signaling pathways without interfering NF-κB and PI3k/Akt signaling pathways. **a**–**c** After pretreatment with 10 μM artemether or DMSO for 6 h, RAW264.7 cells were stimulated with 100 ng/mL RANKL for indicated periods (0, 5, 10, 20, 30, or 60 min) and phosphorylation of p-ERK, p-JNK, p-p38, p-IκBa, p-p65, and p-Akt was determined by western blotting and quantified accordingly. **d**–**g** RAW264.7 cells were pretreated with 10 μM artemether or DMSO for 6 h, and subsequently treated with 100 ng/mL RANKL for 0 or 30 min. Influence of artemether on phosphorylation of p-MEK1/2, p-MKK3/6, and p-MKK7 were determined by western blotting and quantified. **e**, **h** RAW264.7 cells were pretreated with 10 μM artemether or DMSO for 6 h, and subsequently treated with 100 ng/mL RANKL for 0, 1, or 5 min. Phosphorylation of the upstream kinase of MKKs, TAK1, was determined by western blotting and quantified. **f**, **i** BMMs were cultured in induction medium with or without 10 μM artemether for 0, 1, 3, and 5 days, the expression of c-Fos and NFATc1 was determined by western blotting. All the data were confirmed by three repeated tests. Data were expressed as mean ± SD. *p < 0.05 and **p < 0.01 vs. the control group

Several signaling pathways were reported to be involved in the differentiation of BMM to osteoclast, mainly, including NF-κB, MAPKs, and PI3k/Akt^22,23^. To get further insights into the mechanism underlying artemether-induced inhibition of osteoclast differentiation, we investigated the influence of artemether on RANKL-induced pathways using western blotting. After pretreatment with or without artemether for 6 h, RAW264.7 cells were stimulated with 100 ng/ml RANKL for indicated times (0, 5, 10, 20, 30, and 60 min) to observe the activation of above mentioned signaling pathways. The results showed that artemether suppressed the activation of MAPK subfamilies, including ERK, JNK, and p38 (Fig. 4a). In contrast, artemether showed no drastic inhibition on NF-κB (IκBα and p65) and PI3k/Akt pathways (Fig. 4b). In control group, the phosphorylation of ERK, JNK, and p38 were peaked within 30 min, 30 min, and 20 min after RANKL stimulation, respectively. However, pretreatment with artemether significantly decreased the trend of phosphorylation within these signaling pathways (Fig. 4c).

To further explore whether artemether directly blocked the phosphorylation of MAPKs, activation of upstream regulators was determined by western blotting. Results indicated that RANKL-induced phosphorylation of MAP kinase kinase 1/2 (MEK1/2), MAP kinase kinase 3/6 (MKK3/6), and MAP kinase kinase 7 (MKK7) in RAW264.7 were all inhibited after treatment of artemether (10 μM) (Fig. 4d, g). Then we explored the effect of artemether on phosphorylation of transforming growth factor β-activated kinase 1 (TAK1), which is an upstream kinase of MKKs^24^. However, phosphorylation of TAK1 was not significantly attenuated in presence of artemether (Fig. 4e, h).

We next examined whether artemether altered the expression of c-Fos and NFATc1 following RANKL stimulation, as activation of MAPK pathways subsequently leads to the induction and activation of c-Fos and NFATc1, which are key transcription factors for osteoclast differentiation^25^. BMMs were cultured in the absence or presence of artemether (10 μM) for 0, 1, 3, and 5 days in differentiation medium. Results of western blotting indicated that artemether treatment significantly downregulated the expression of c-Fos and NFATc1 in BMMs after RANKL stimulation (Fig. 4f, i).

### Artemether protected against LPS-induced osteolytic bone loss

**Fig. 5:**
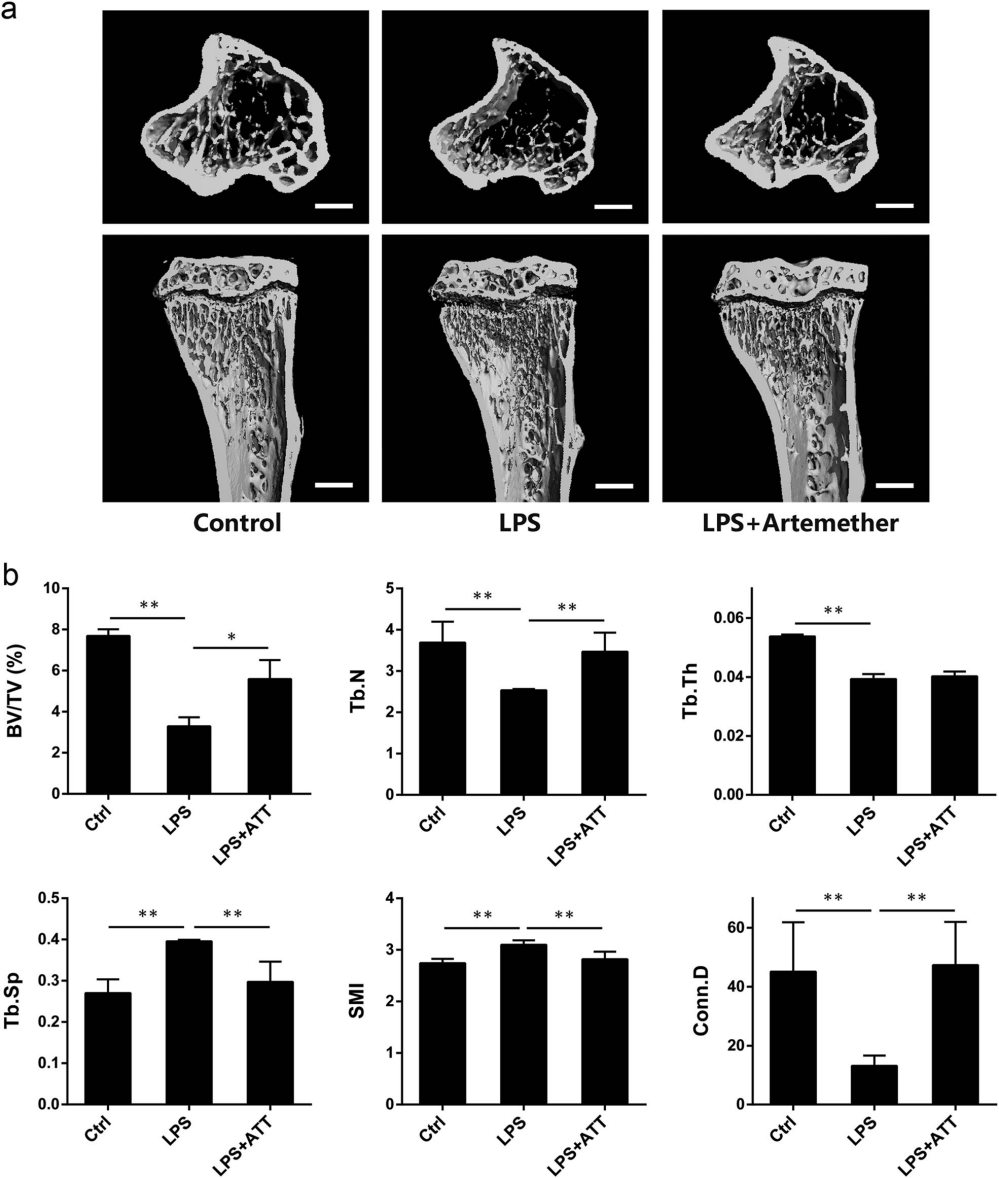
Artemether mitigated inflammatory bone erosion in a murine tibial model of LPS-induced inflammatory bone loss. **a** Representative micro-CT reconstruction images of three groups. **b** Micro-CT analyses of region of interest. All the data were confirmed by three repeated tests. The data were expressed as mean ± SD. *p < 0.05 and **p < 0.01 vs. the control group. Scale bar = 500 μm

In order to explore the antiresorptive capability of artemether on skeletal system, an in vivo model of LPS-induced inflammatory bone loss was adopted^5^. For this, 8-week-old male C57BL/6 mice received intraperitoneal injection of DMSO, LPS, or LPS+Artemether for 8 days. On day 8 after the first LPS administration, tibias was collected and analyzed by micro-CT. The three-dimensional micro-computed tomographic analysis revealed extensive trabecular bone loss in LPS group as compared with control (Fig. 5a). And morphometric analysis revealed pronounced reduction of BV/TV, Tb.N, Tb.Th, and Conn.D, and marked increase of Tb.Sp and SMI after LPS administration. On the other hand, artemether treatment effectively prevented the osteolytic bone loss and the reduction in bone volume. Moreover, increased Tb.Sp and SMI was also reversed (Fig. 5b).

**Fig. 6:**
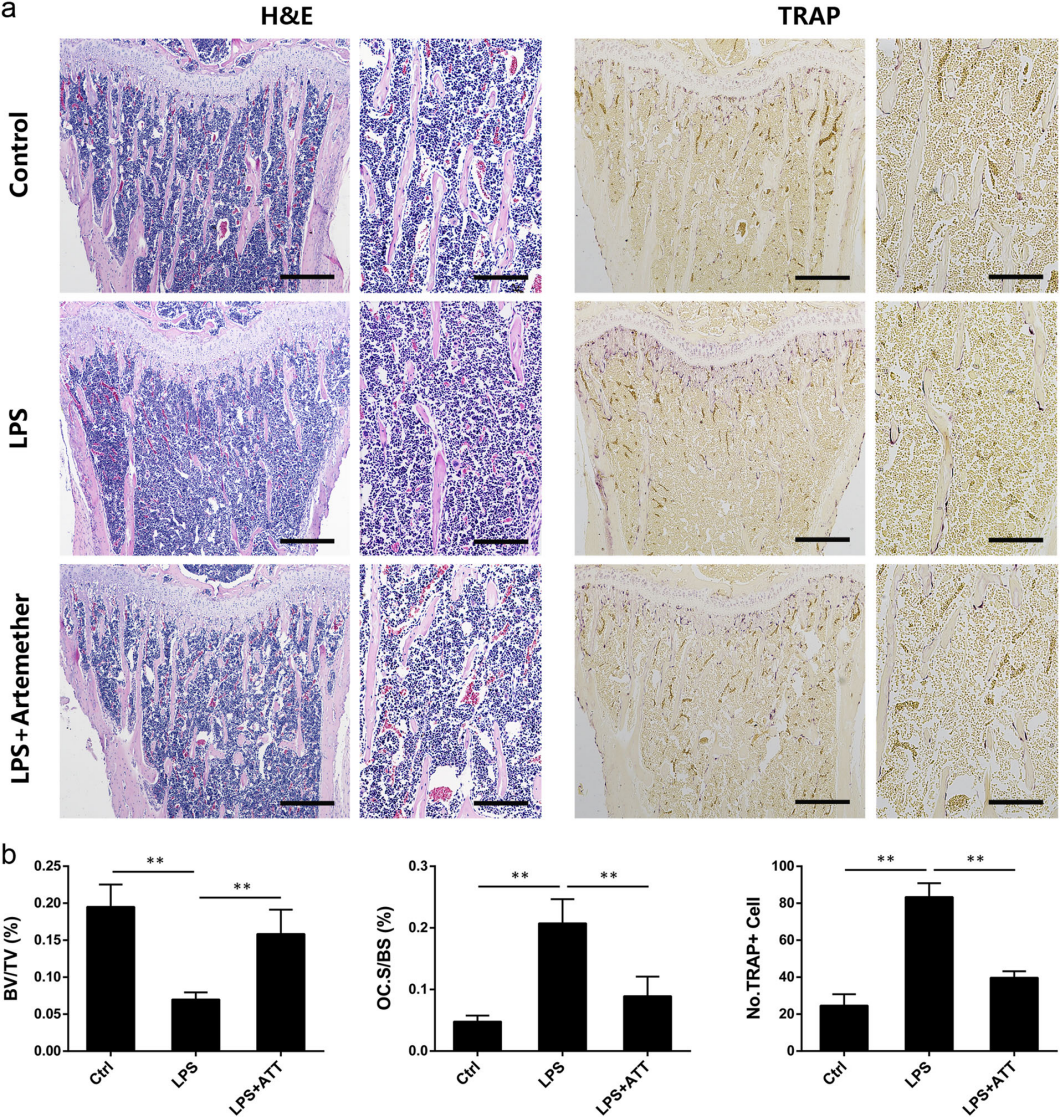
Histological evaluation of the effects of artemether on LPS-induced inflammatory bone loss. **a** Representative images of decalcified bone stained with H&E and TRAP. **b** Quantitative analyses of bone volume/total volume (BV/TV), osteoclast surface/bone surface (Oc.S/BS), number of TRAP-positive cells. All the data were confirmed by three repeated tests. The data were expressed as mean ± SD. **p < 0.01 vs. the control group. Scale bar = 400 μm for general view, and scale bar = 160 μm for magnified view

Histological analyses further confirmed the protective effect of artemether on LPS-induced osteolysis. Consistent with the results of micro-CT, there was an increase in BV/TV in the LPS+Artemether group when compared with LPS group. Additionally, osteoclast surface/bone surface (Oc.S/BS) and number of TRAP-positive cells were significantly reduced in the LPS+Artemether group with concomitant reduction in inflammatory osteolysis when compared with the LPS group (Fig. 6a, b). These results demonstrated the protective effects of artemether against LPS-induced inflammatory bone loss in vivo.

## Discussion

Total joint arthroplasty is a successful procedure for end-stage joint disease. The major reason for the long-term failure is aseptic loosening owning to periprosthetic osteolysis^26^, which is mainly triggered by an inflammatory response to wear particles generated from implant. Conservatively, aseptic loosening occurs in the absence of clinical or laboratory signs of bacterial infection^27^. But there have been increasing evidence of positive bacterial isolates identified in patients with clinically absent infection^28^. Bacterial endotoxin, which contaminates prostheses, has been identified as a primary contributor to debris-induced osteolysis as it enhances the reactivity of wear particles and increases their proinflammatory effects^4,29^. It can initiate the recruitment of immune cells like monocytes and macrophages and further stimulate the secretion of pro-osteoclastogenic cytokines that are essential to the osteoclast formation, activation, and bone resorption^1,21,30,31^. Osteoclast has long been considered as the major target for therapeutic agents to treat osteolytic bone loss^14^. In this study, we reported that artemether was capable of inhibiting LPS-induced osteolysis in vivo, and the effect was mediated by suppression of the RANKL-induced MAPK (ERK, JNK, and p38) signaling pathways during osteoclast differentiation.

Lipopolysaccharide (LPS) is a membrane component of Gram-negative bacteria and has long been recognized as a potent inducer of the development of osteolytic bone loss^1,31^. Studies have suggested that microbial-associated molecular patterns (MAMPs) could adhere to generated wear debris and cause immunostimulation in arthroplasty cases^32,33^, and this would further lead to a substantial inflammatory reaction, over-reaching the biological effects of the biomaterials themselves^34^. As the most studied MAMP, LPS was used in this study to induce inflammatory bone erosion in a murine tibial model.

Artemether is an artemisinin derivative isolated from Artemisia annua L. and its combination with lumefantrine is one of the first-line antimalarial regimens^35^. It has been demonstrated to have anti-inflammatory effects in experimental rheumatoid arthritis^20^, and in BV2 microglia through inhibition of NF-κB and p38 MAPK signaling^19^. Current evidence suggested that some anti-inflammatory agents could inhibit osteoclastogenesis via suppressing NF-κB and/or MAPK signaling pathways^16,36^. In this regard, the present study was designed to determine whether artemether can exert inhibitory effects on osteoclastogenesis and in a murine model of inflammatory bone loss, and its underlying mechanism.

The central role of osteoclast in inflammatory bone erosion makes it an attractive target for potential therapies^14^. In present study, artemether was proven to be an effective inhibitor of osteoclast formation in vitro as regard to the reduction of the number and area of TRAP-positive multinucleated cells. And we also confirmed that treatment of artemether suppressed the mRNA expression of osteoclast marker genes, including NFATc1, TRAP, V-ATPase d2, CTSK, DC-STAMP, and MMP-9. Result of in vitro resorption pit was consistent with the inhibition of artemether on osteoclast formation. Furthermore, artemether administration significantly reduced the number of TRAP-positive cells in vivo when compared with the LPS group. The relevant data suggested that the protective effect of artemether on inflammatory bone loss was based on its inhibition of osteoclast formation and function.

Then we investigate the underlying mechanism of artemether-induced inhibition on osteoclast formation. RANKL-induced NF-κB and PI3K/Akt activation is important for osteoclast differentiation and function^16,37,38^, however, the two pathways seemed to be not affected in the presence of artemether. Besides, the MAPK signaling pathways also play pivotal roles in osteoclast formation^39^. Our results demonstrated that artemether exhibited potent inhibition on osteoclast formation and bone resorption via suppression of RANKL-induced MAPK (ERK, JNK, and p38) signaling pathways, which was in line with the therapeutic effect of artemether on osteolytic bone loss observed in vivo. Previous studies have revealed the crucial roles of MAPK pathways in osteoclast formation and inflammatory bone loss^1,15,39,40^. Subfamilies of MAPK pathway (ERK, JNK, and p38) are phosphorylated preferentially by MEK1/2, MKK7, and MKK6, respectively. Among this, phosphorylation of ERK would later promote the induction of c-Fos, which is an essential member of AP-1 transcription factor complex^41^, and this is of vital importance to NFATc1 induction and osteoclast differentiation. In addition, the broad spectrum of inhibition on the MAPK subfamilies suggests that artemether might target an common upstream kinase or phosphatase in MAPK pathway, which is testified by results of western blotting that artemether mainly targets the MKKs. Activation of MAPKs would synergistically induce the autoamplification of NFATc1^42^. NFATc1 is a well-characterized master transcription factor required for osteoclastogenesis and regulatoin of marker genes, including TRAP, V-ATPase d2, CTSK, DC-STAMP, and MMP-9, which are essential for osteoclast precursor fusion and osteoclastic bone resorption^7,43–45^. Along with the attenuated activation of MAPK pathways, the transcriptional activity and protein induction of NFATc1 was reduced markedly in the presence of artemether. And the expression of marker genes was also dampened in a dose-dependent manner. These results suggest that inhibition of artemether on osteoclastogenesis could be partly attributed to the mitigation of MAPK signaling cascades and thereafter the impaired NFATc1 induction. Furthermore, the phosphorylation of MEK1/2, MKK3/6, and MKK7, but not TAK1, was suppressed by artemether. To sum up, these results indicate artemether acts to suppress the activation of MKKs/MAPKs/NFATc1 signaling during osteoclastogenesis without interfering NF-κB and PI3K/Akt activation.

LPS is capable of activating inflammatory cells, promoting secretion of proinflammatory cytokines^46^, and finally inducing osteoclast precursors infusion and osteoclastic bone erosion^47^. A model of LPS-induced inflammatory bone loss was successfully set up by stimulating distinct bone erosion at secondary spongiosa of murine tibia. As expected, artemether treatment significantly mitigated the severity of bone erosion of the model, illustrated by an increase in BV/TV, Tb.N, and Conn.D, and a decrease of Tb.Sp and SMI in micro-CT scanning. Similarly, bone histomorphometry further confirmed that artemether treatment could inhibit LPS-induced inflammatory bone loss in vivo accompanied by a decrease of the TRAP-positive cells. All these evidence suggested that artemether had a therapeutic potential for treatment of inflammatory bone loss.

In spite of the novel findings in our study, there are several limitations merit discussion. First, the interaction between bone resorption and osteogenesis is complex and osteoblastic bone formation also plays an important role in treatment of osteolysis. However, we have focused on the effect and mechanism of artemether on osteoclastic bone erosion in present study. In vivo study demonstrated that intraperitoneal administration of artemether strongly alleviated the bone loss and emergence of TRAP-positive cells induced by LPS. As the undesirable status was not completely reversed, the inhibition of bone destruction by artemether was presumed to be mainly owning to its suppression on osteoclastogenesis. Further studies are needed to address its effect on osteoblast and the possible mechanism. Second, initiating agent of osteolysis other than LPS should be tested to further confirm the beneficial effect of artemether.

In summary, findings of the study demonstrated that artemether inhibited osteoclastogenesis and osteoclast function in vitro and LPS-induced inflammatory bone loss in vivo. We have clarified that it worked via suppression of the MAPK signaling pathways and the downstream factors NFATc1. These results suggested that artemether might serve as a potential therapeutic regimen for osteoclast-related disorders.

## Materials and Methods

### Reagents

Fetal bovine serum (FBS) and alpha modification of Eagle medium (α-MEM) were purchased from Gibco (Sydney, Australia). The cell counting kit (CCK-8) was obtained from Dojindo Molecular Technologies (Kumamoto, Japan). Recombinant murine M-CSF and recombinant murine RANKL were purchased from PeproTech (Rocky Hill, USA). Primary antibodies against ERK, phospho-ERK (Thr202/Tyr204), JNK, phospho-JNK (Thr183/Tyr185), p38, phospho-p38 (Thr180/Tyr182), p65, phospho-p65 (Ser536), IκBα, phospho-IκBα (Ser32), Akt, phospho-Akt(Ser473), transforming growth factors-β-activated kinase 1 (TAK1), phosphor-TAK1 (Ser412), MAP kinase kinase 1/2 (MEK1/2), phospho-MEK1/2 (Ser217/221), MAP kinase kinase 3/6 (MKK3/6), phospho-MKK3(Ser189)/6(Ser207), MAP kinase kinase 7 (MKK7), phospho-MKK7 (Ser271/Thr275), NFATc1/NFAT2, β-actin, and HRP-linked secondary antibodies against mouse or rabbit IgG were purchased from Cell Signaling Technology (Danver, MA, USA). Primary antibody against c-Fos was purchased from Abcam (Cambridge, MA). The TRAP staining kit, DMSO, and LPS were purchased from Sigma-Aldrich (St. Louis, USA). Artemether was purchased from Tauto Biotech (Shanghai, China).

### Bone marrow-derived macrophages isolation and osteoclast culture

Bone marrow-derived macrophages (BMMs) were prepared as previously described^48^. Briefly, bone marrow cells were isolated from long bones of 6-8-week-old C57BL/6 mice and differentiated into BMMs in complete α-MEM medium with 25 ng/ml M-CSF. Next, BMMs were seeded (8 × 10^3^ cells per well) into 96-well plate and cultured with complete α-MEM medium containing M-CSF (25 ng/ml) and RANKL (100 ng/ml) for 5–6 days with various concentrations of artemether (0, 1.25, 2.5, 5, 7.5, 10 μM). Cells were cultured in an incubator at 37 °C with 5% CO_2_ atmosphere and the medium was refreshed every second day until mature osteoclasts had formed. Then cells were washed by PBS twice and fixed with 4% paraformaldehyde and stained for TRAP according to manufacturer’s instruction. TRAP-positive cells containing three or more nuclei were counted as mature osteoclasts. The number of osteoclasts and their spread area were measured with ImageJ software (NIH, MD, USA).

### Cell viability and cytotoxicity assay

The effect of artemether on BMMs viability was determined by CCK-8 assay^36^. The BMMs were seeded into 96-well plate at s density of 8 × 10^3^ cells per well and cultured in complete α-MEM medium containing 25 ng/ml M-CSF and serial concentration of artemether (0, 10 nM, 100 nM, 1 μM, 10 μM, 100 μM, and 1 mM) for 72 h. Then the medium was removed and cells were cultured in 100 μL α-MEM medium with M-CSF and 10% CCK-8 for 4 h at 37 °C. The optical density (OD) value at wavelength of 450 nm, which is directly proportional to cell viability/proliferation, was measured by an ELX808 absorbance microplate reader (BioTek, Winooski, VT, USA).

### Analysis of apoptosis by flow cytometry

BMMs were exposed to artemether (0, 2.5, 5, 10 μM) for 72 h. After washing with precooled PBS twice, cells were suspended in 500 μL binding buffer and stained with Annexin V-PE and 7-amino-actinomycin (7-AAD) for 15 min at room temperature (RT) in the dark. Then cells were excited at 488 nm and signals from 10,000 cells were acquired at 585/42 nm and 702/64 nm by FACS Canto II (BD, Triangle, NC, USA). Results were analyzed with FACSDiva 6.1.3 software and expressed as the percentage of apoptotic cells.

### RNA isolation, reverse transcription, and quantitative PCR

Total RNA was isolated using RNAiso reagent (TaKaRa, Dalian, China) and quantified by NanoDrop 2000 (Thermo Fisher Scientific, Waltham, MA, USA) with absorbance at 260 nm. Total RNA (≤1000 ng) was reverse-transcribed into cDNA in a reaction volume of 20 μL according to the manufacturer’s instructions (Takara, Dalian, China). Real-time PCR was performed with SYBR^®^ Premix Ex Taq^TM^ II (TaKaRa) on the ABI StepOnePlus System (Applied Biosystems, Warrington, UK). Beta-actin was amplified as housekeeping gene, and all the reactions were repeated in triple. The murine primer sequences of TRAP, NFATc1, V-ATPase d2, CTSK, dendritic cell-specific transmembrane protein (DC-STAMP), matrix metalloproteinase 9 (MMP-9), and β-actin were presented in Table 1. The following cycling conditions were used: 95 °C for 30 s and 40 cycles of denaturation at 95 °C for 5 s and amplification at 60 °C for 30 s. The relative target gene expression levels were calculated using the 2 ^−△△Ct^ method.

**Table 1:** Primers used for quantitative PCR

Gene	Forward (F) and reverse (R) primer sequence (5′–3′)
TRAP	F: CACTCCCACCCTGAGATTTGT
	R: CCCCAGAGACATGATGAAGTCA
NFATc1	F: CCGTTGCTTCCAGAAAATAACA
	R: TGTGGGATGTGAACTCGGAA
V-ATPase-d2	F: AAGCCTTTGTTTGACGCTGT
	R: TTCGATGCCTCTGTGAGATG
CTSK	F: CTTCCAATACGTGCAGCAGA
	R: TCTTCAGGGCTTTCTCGTTC
DC-STAMP	F: AAAACCCTTGGGCTGTTCTT
	R: AATCATGGACGACTCCTTGG
MMP-9	F: CAAAGACCTGAAAACCTCCAA
	R: GGTACAAGTATGCCTCTGCCA
β-actin	F: TCTGCTGGAAGGTGGACAGT
	R: CCTCTATGCCAACACAGTGC

### Osteoclast-mediated bone resorption pit assay

The bone resorption pit assay was conducted as previously described^36^. BMMs were seeded onto bovine bone discs at a density of 2.4 × 10^4^ cells/cm^2^. After adherence, cells were treated with M-CSF (25 ng/ml), RANKL (100 ng/ml), and artemether (0, 2.5, 5, and 10 μM) until mature osteoclasts formed. Then adhered cells were removed by gentle brushing. Resorption pits were visualized by scanning electron microscope (Hitachi S-3700N) and the percent resorbed areas were quantified using ImageJ software. Three random view fields of the discs were selected for analysis and the similar experiments were repeated in triple.

### Western blot analysis

RAW264.7 cells were seeded in 6-well plates at a density of 5 × 10^5^ cells per well. After exposure to vehicle or artemether for 6 h, the cells were stimulated with RANKL (100ng/ml) for indicated time. BMMs were cultured with 0 or 10 μM artemether, in the presence of M-CSF (25 ng/ml) and RANKL (100 ng/ml) for 0, 1, 3, and 5 days. Total cellular proteins were extracted from cultured cells using RIPA lysis buffer supplemented with protease inhibitor and phosphatase inhibitor cocktail (Fude Biological Technology Co. Ltd, Hangzhou, China). Lysates were incubated on ice for 30 min and cleared by centrifugation at 14,000 r.p.m. for 15 min at 4 °C and supernatants containing proteins were collected. The concentration of protein was calibrated with BCA protein assay kit (Boster Biological Technology Co. Ltd, Wuhan, China). Equal amounts of protein lysates were separated using 10% sodium dodecyl sulfate-polyacrylamide gel electrophoresis (SDS-PAGE) and electroblotted onto polyvinylidene difluoride (PVDF) membrances (Millipore, Shanghai, China). The membranes were blotted with 5% BSA in TBS-0.1% Tween (TBST) for 1 h and then probed with primary antibodies at 4 °C overnight. The membranes were incubated with horseradish peroxidase-linked secondary antibodies for 2 h at 4 °C, and antibody reactivity was detected using the enhanced chemiluminescent detection reagent (Millipore, Shanghai, China) in a Bio-Rad XRS chemiluminescence detection system (Bio-Rad, Hercules, CA, USA). Signal intensity of the bands was quantified by Quantity One software.

### In vivo murine model of LPS-induced bone loss

All the procedures were approved by the Animal Care and Use Committee of the Second Affiliated Hospital of Zhejiang University School of Medicine. The methods were carried out in the range of approved guidelines of the university. Eight-week-old male C57BL/6 mice were provided by Shanghai SLAC Laboratory Animal Co. Ltd. Then they were randomized into three groups with six mice in each: DMSO-treated (Control group), LPS (5 μg/g)-treated (LPS group), and LPS+Artemether (10 μg/g) (LPS+Artemether group)-treated groups. Artemether was dissolved in DMSO and LPS was dissolved in phosphate buffered saline (PBS). LPS was administrated intraperitoneally on day 1 and 4. One day before injection of LPS and subsequently on every other day for up to 8 days till the end of the experiment. All mice were killed by cervical dislocation, and the tibias were collected and scanned by micro-CT (μCT 100; SCANCO Medical, Bassersdorf, Switzerland). Data of bone mineral density (BMD), structure model index (SMI), connectivity density (Conn.D), bone volume/tissue volume (BV/TV), trabecular thickness(Tb.Th), trabecular number (Tb.N), and trabecular separation (Tb.Sp) were collected to evaluate the trabecular microstructure of the tibias. All the tibias were fixed in 4% paraformaldehyde for 1 day and decalcified in 10% EDTA (pH 7.4) for 4 weeks. After decalcification, specimens were embedded and sectioned to slices of 3 um thickness, and stained with H&E and TRAP for histologic examination.

### Statistical analysis

The data were expressed as mean ± SD. All the experiments were carried out independently at least 3 times. Statistical analysis was performed by unpaired Student’s *t* test using SPSS 19.0 software (SPSS Inc., USA). Any *p* values < 0.05 indicated a significant difference between treated and control groups.
